# Dynamic Disturbance Processes Create Dynamic Lek Site Selection in a Prairie Grouse

**DOI:** 10.1371/journal.pone.0137882

**Published:** 2015-09-22

**Authors:** Torre J. Hovick, Brady W. Allred, R. Dwayne Elmore, Samuel D. Fuhlendorf, Robert G. Hamilton, Amber Breland

**Affiliations:** 1 School of Natural Resource Sciences—Range Program, North Dakota State University, Fargo, North Dakota, United States of America; 2 College of Forestry and Conservation, University of Montana, Missoula, Montana, United States of America; 3 Natural Resource Ecology and Management, Oklahoma State University, Stillwater, Oklahoma, United States of America; 4 Tallgrass Prairie Preserve, The Nature Conservancy, Pawhuska, Oklahoma, United States of America; 5 Dahomey National Wildlife Refuge, United States Fish and Wildlife Service, Boyle, Mississippi, United States of America; Oregon State University, UNITED STATES

## Abstract

It is well understood that landscape processes can affect habitat selection patterns, movements, and species persistence. These selection patterns may be altered or even eliminated as a result of changes in disturbance regimes and a concomitant management focus on uniform, moderate disturbance across landscapes. To assess how restored landscape heterogeneity influences habitat selection patterns, we examined 21 years (1991, 1993–2012) of Greater Prairie-Chicken (*Tympanuchus cupido*) lek location data in tallgrass prairie with restored fire and grazing processes. Our study took place at The Nature Conservancy’s Tallgrass Prairie Preserve located at the southern extent of Flint Hills in northeastern Oklahoma. We specifically addressed stability of lek locations in the context of the fire-grazing interaction, and the environmental factors influencing lek locations. We found that lek locations were dynamic in a landscape with interacting fire and grazing. While previous conservation efforts have treated leks as stable with high site fidelity in static landscapes, a majority of lek locations in our study (i.e., 65%) moved by nearly one kilometer on an annual basis in this dynamic setting. Lek sites were in elevated areas with low tree cover and low road density. Additionally, lek site selection was influenced by an interaction of fire and patch edge, indicating that in recently burned patches, leks were located near patch edges. These results suggest that dynamic and interactive processes such as fire and grazing that restore heterogeneity to grasslands do influence habitat selection patterns in prairie grouse, a phenomenon that is likely to apply throughout the Greater Prairie-Chicken’s distribution when dynamic processes are restored. As conservation moves toward restoring dynamic historic disturbance patterns, it will be important that siting and planning of anthropogenic structures (e.g., wind energy, oil and gas) and management plans not view lek locations as static points, but rather as sites that shift around the landscape in response to shifting vegetation structure. Acknowledging shifting lek locations in these landscapes will help ensure conservation efforts are successful by targeting the appropriate areas for protection and management.

## Introduction

Fire and herbivory were historically dynamic disturbances that played critical roles in the establishment and maintenance of grasslands and savannas [1–4]. In addition to the individual effects of these disturbances, their interactions are important in shaping the structure and function of grassland ecosystems [5–7]. Fire and grazing processes in grasslands were historically intermittent and patchy in nature, creating spatial patterns that varied greatly in connectivity, size, shape, and structure [8]. These complex processes have been simplified through time and have resulted in more uniform landscapes [9]. Alteration of fire regimes, intensified agriculture, and the replacement of roaming native large herbivores with fenced and stationary domestic livestock have all contributed to the homogenization of grasslands [9–11]. This simplification likely altered the behavior of native fauna as landscape spatial patterning can affect the distribution, movement, and persistence of a species [12–13]. Furthermore, investigation of species habitat selection in landscapes that lack dynamic processes has likely contributed to ineffective conservation and management paradigms focused on homogeneity [14]. As an example, species diversity for a variety of taxa (i.e., insects, birds, and mammals) and ecosystem processes such as plant and bird community stability, fire behavior, and hydrology can all be increased through managing complex landscapes for heterogeneity [14].

Understanding the consequences of spatial heterogeneity for animal populations has long been a central theme in landscape ecology [12]. Research suggests that changes in large scale land-use patterns and fragmentation of grassland landscapes have been associated with the decline of many imperiled wildlife populations [14–16]. The widespread decline of grassland birds may be viewed as a consequence of the deviation of current grasslands from the structurally varying systems that once occurred [17–18]. Because grassland bird species evolved within the context of a shifting grassland mosaic, some species were restricted to areas with specific plant composition and structure created under spatially and temporally distinct disturbance regimes [17, 19]. The elimination of heterogeneity from most remaining grasslands has decreased habitat availability and suitability for many grassland bird species, especially those requiring either undisturbed or heavily disturbed areas such as Henslow’s Sparrow (*Ammodramus henslowii*) or Upland Sandpiper (*Bartramia longicauda*), respectively [18, 20–21]. Furthermore, some grassland obligate fauna may require heterogeneous landscapes to meet various life history requirements and the loss of dynamic disturbance processes can negatively affect populations [22–23]. One such species, the Greater Prairie-Chicken (*Tympanuchus cupido*; hereafter, prairie-chicken), has experienced substantial population declines across its distribution [16, 24].

Prairie-chickens were once common throughout parts of the United States and Canada [25–26], but in recent times have undergone population declines near 70% in the core of their distribution [22, 27]. Commonly referred to as an umbrella species for the tallgrass prairie ecosystem [28–29], targeted conservation efforts for prairie-chickens have become widespread. Conservation is frequently focused in areas proximate to prairie-chicken leks—areas used for communal courtship displays [30–31]. Data collected from lek counts are a common source for establishing indices of prairie-chicken abundance and population trends. Because lek sites are frequently the focus for conservation planning [24–25, 31], it is important that researchers and managers understand the factors that affect both lek site selection and stability. In this context, we refer to stability as the likelihood that a lek will occur in the same geographic location from one year to the next, rather than the number of individuals present from year to year which can greatly fluctuate relative to annual weather variability.

Previous prairie-chicken research has predominantly occurred in landscapes lacking critical disturbance regimes (i.e., fire, and fire/grazing interactions). As a result, these studies report lek sites as static or permanent and describe “traditional leks”—those that occur in the same location each year—as being associated with larger patches of grassland and in areas with greater distance from trees [32–34]. This binary classification of leks can create confusion as practitioners and land managers shift towards management regimes that restore disturbance processes. Undoubtedly, traditional and stable lek locations are an accurate description of lek site selection in static landscapes, and this permanency of leks has been a justification for identifying preferred prairie-chicken habitat that dictates landscape management decisions [33]. The problem with this static view of habitat selection by prairie-chickens is that it is dictated by the landscape level management, and may be an artifact of altered grassland processes rather than the inherent species behavior.

Present-day grassland management practices that restore the interactive effects of fire and grazing are increasingly being used to create grassland heterogeneity and conserve biodiversity [18, 35–37]. As this restoration of heterogeneity affects the diversity, survival, and patch occupancy of numerous grassland birds [18, 21, 38–39], it is reasonable to assume that prairie-chicken habitat selection will also be affected. Shifts in lek locations or attendance have resulted from fire [32] but the mechanisms are unknown. Additional research indicates that 95% of prairie-chicken females initiated nests within 2 km of lek locations [23]. Moreover, research investigating lek site selection indicates that males attempt to select areas that maximize their potential for encountering females (i.e., the hotspot hypothesis; [40]). As a consequence, it is likely that lek site selection by prairie-chickens will track the shifting grassland mosaic in response to changes in biomass and associated nest site suitability. Grasslands with shifting mosaics that result from the interaction of fire and grazing are more structurally diverse than grasslands managed with fire or grazing alone, and these diverse grasslands may be more similar to pre-settlement conditions under which prairie-chickens evolved [18]. Therefore, it is important that results from research in landscapes managed for homogeneity are not uniformly applied to prairie-chicken lek site selection in all landscapes, especially those with restored dynamic processes. Further, if managers aim to restore high-functioning tallgrass prairie by incorporating dynamic disturbance processes, then a static view of lek locations may not be adequate.

We examined prairie-chicken lek site selection patterns in a landscape with restored heterogeneity as a result of interacting fire and grazing processes. Heterogeneity in this context exists as large herbivores are attracted to recently burned patches while avoiding areas that have gone unburned in multiple years [35]. The result is a shifting structural mosaic with short vegetation that is recently burned and grazed, intermediate vegetation structure that was grazed and burned in recent years, and tall vegetation structure that has gone multiple years without being burned or grazed [9, 35]. We hypothesized that shifting grassland structure would create annual movements in prairie-chicken lek location patterns as male prairie-chickens attempt to place lek locations adjacent to nearby nesting cover to maximize female encounters. We examined 21 (1991, 1993–2012) years of lek locations to determine 1) the stability of lek locations in the context of the fire-grazing interaction and 2) the environmental factors influencing lek site selection and movements.

## Methods

### Study site

**Fig 1:**
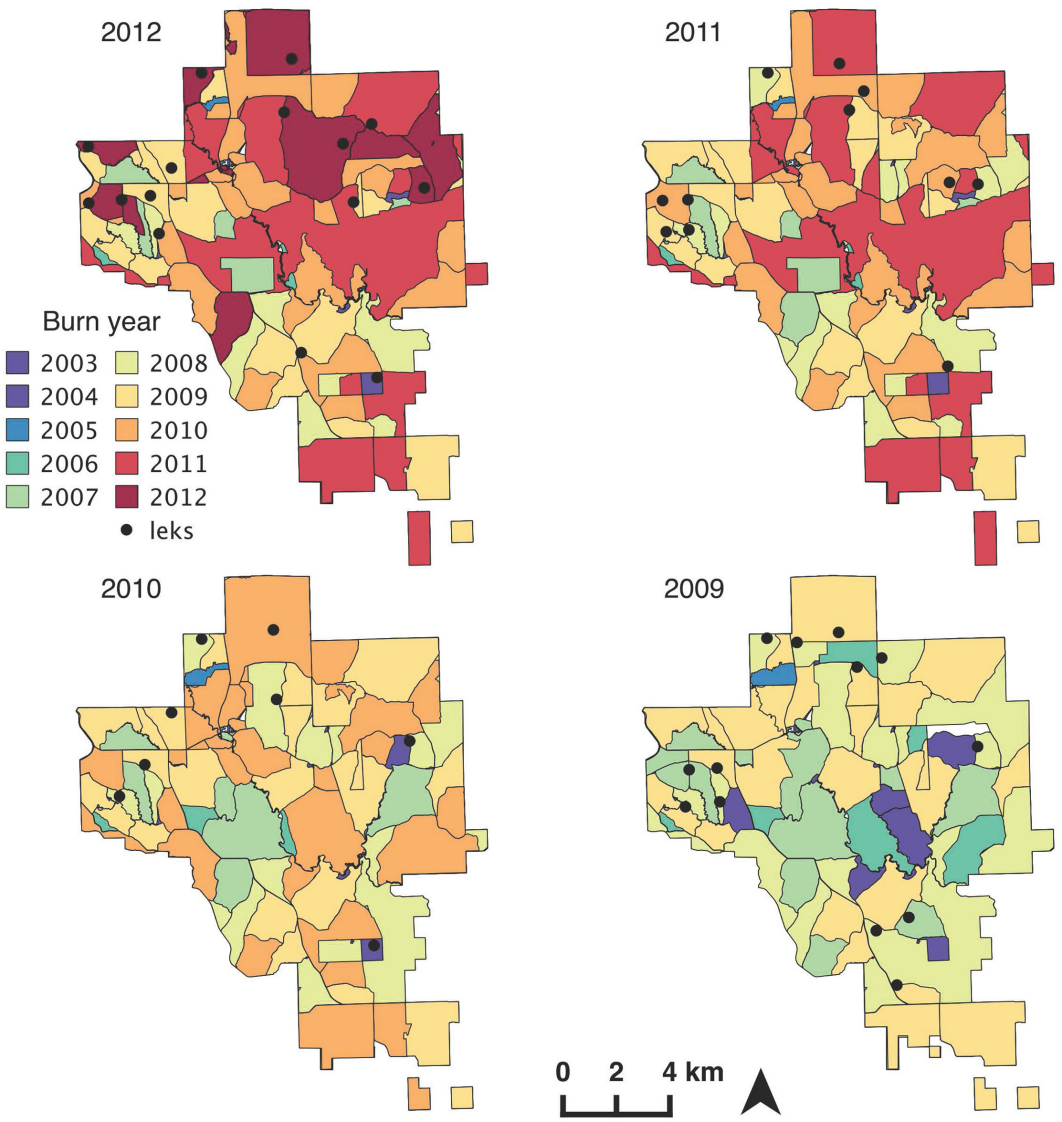
Creating spatial and temporal heterogeneity. Example of the fire regime used to restore heterogeneity at The Nature Conservancy’s Tallgrass Prairie Preserve, OK, USA. Each color represents a different year of fire (seasons not shown) and black dots indicate leks present that year. Because of the fire-grazing interaction, vegetation structure varies across patches with different times post fire which creates structural heterogeneity across the landscape.

We investigated prairie-chicken lek location dynamics at The Nature Conservancy’s ~16,000 ha Tallgrass Prairie Preserve (hereafter, the preserve). The preserve was in Osage County, Oklahoma (36°50’ N, 96°25’ W) and represents the southernmost extent of the remaining Flint Hills tallgrass prairie. Management at the preserve is focused on conserving biodiversity through the restoration of grassland heterogeneity using spatially discrete fires (hereafter, patches) and focused grazing by native bison (*Bison bison*) and domestic cattle (*Bos taurus*) [35, 41]. On average, one-third of the preserve is burned each year with around 80% burned during the dormant season (spring and fall) and the remaining 20% burned during the growing season (late summer) (Fig 1). The plant community is primarily tallgrass prairie with small patches of cross timbers forest along large streams. Dominant grasses include big bluestem (*Andropogon gerardii* Vitman), little bluestem (*Schizachyrium scoparium* (Michx.) Nash), switchgrass (*Panicum virgatum* L.), and indiangrass (*Sorghastrum nutans* (L.) Nash) and dominant forbs at the preserve include ironweed (*Veronia spp*.), milkweed (*Asclepias spp*.), and ashy sunflower (*Helianthus mollis*). Cross timbers vegetation is dominated by post oak (*Quercus stellata* Wang.) and blackjack oak (*Q*. *marilandica* Münchh) [42]. The climate is temperate with hot summers and moderate levels of precipitation. The long-term average precipitation (1996–2010) for April-August is 540 mm and long term average high temperature for July is 36.9 degrees Celsius [23].

### Data Collection

Lek locations were identified at the preserve each year through systematic searches that covered all grassland dominated plant communities. Searches took place during April from 1991–2012 on days with winds <20 km^-hr^, no precipitation, and minimal cloud cover. Observers started one half hour prior to sunrise and concluded within three hours of sunrise. When leks were located, observers flushed displaying males to count the total number of individuals (males and females if present) and record the coordinates of the lek. Most frequently, the NAD 1983 zone 14 coordinate system was used on GPS units. However, in some circumstances the use of aerial imagery and a familiarity with the working landscape allowed nature conservancy employees to accurately mark images with locations that could be digitized into GIS software—reducing the error associated with changes in technology over time. In all cases, an estimate of the center point was used to record the coordinates. All data collected was on land owned and managed by The Nature Conservancy and no permits were required because there was no handling of wildlife.

### Data analysis

**Fig 2:**
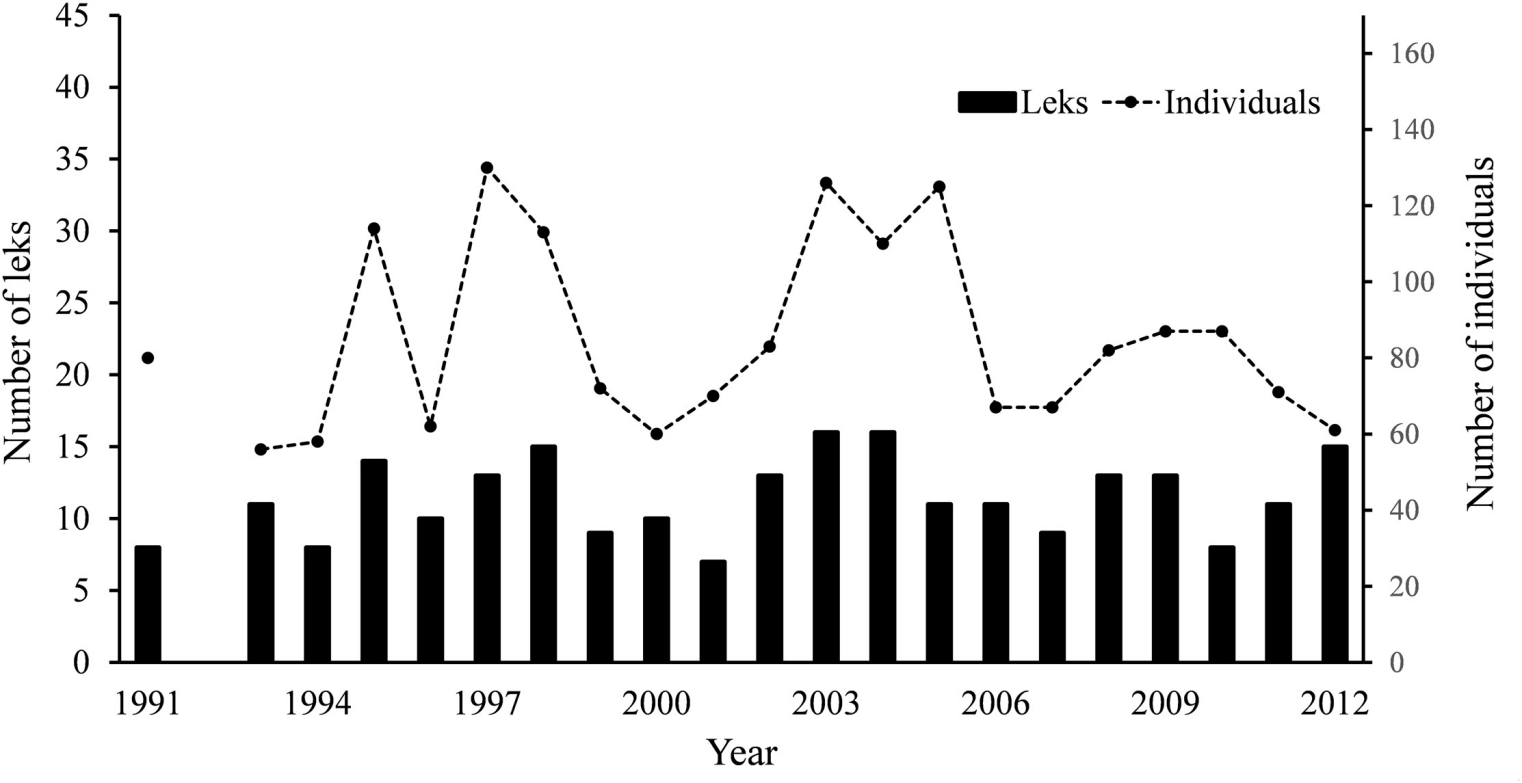
Lek trends. Annual number of leks observed and individuals counted at the The Nature Conservancy’s Tallgrass Prairie Preserve over 21 years (1991, 1993–2012).

We used a two phase approach to determine if lek locations were static or dynamic (objective one). We first calculated the distance of each lek location to the closest lek location of the following year. Small differences between lek locations from successive years would indicate that lek locations were relatively static and permanent, occurring at the same site each year; greater distances would indicate that lek locations were more dynamic and temporary, moving around the landscape. While it is possible that “false” lek movements could be recorded as a result of meta-populations going extinct and then recolonizing, a linear regression assessing the trend of this population shows it has been relatively stable across the 21-year study period and that the number of leks on the landscape have been stable (population trend slope = -0.098, r^2^ = 0.0006; lek trend slope = 0.093, r^2^ = 0.043) (Fig 2). We also addressed this by accounting for potential “drift” movements in leks (and error that could be associated with GPS locations or equipment) by buffering each lek location with a 100, 250, and 500-m radius and calculating the distance of each buffered lek location to the closest buffered lek location of the following year. These buffer sizes represent liberal to more conservative sizes to evaluate whether or not a lek location occurred in the same geographic location each year and have no relevance as to how far individual birds may move, as we did not track individuals. If buffers overlapped within any size class, the lek location was classified as static. We determined the proportion of lek locations that moved each year for each buffer size by dividing the number of leks locations that moved by the total number of lek locations for that year. Additionally, we classified lek locations as moved (1) or present (0) and calculated a logistic regression using lek size as a predictive variable to assess the influence of the number of individuals present at a lek location on the likelihood a lek would be at that same location the following year.

To determine how environmental factors influenced lek locations and movements (objective two), we estimated resource selection functions using logistic regression models [43]. We chose environmental factors that were previously reported to influence lek locations [32–34], or relevant to addressing our objectives and hypotheses. Our environmental variables included: elevation and slope (United States Geological Survey National Elevation Dataset), distance to (fire) patch edge, road density calculated as the length of roads within a 2 km buffer divided by the total area in the 2 km buffer, distance to nearest road, time since fire, woody cover calculated as the area covered by trees within the 2 km buffered divided by the areas of the 2 km buffer, and the interaction of time since fire with distance to patch edge. We did not use a separate grazing variable because grazing is highly dependent on fire in this landscape and time since fire largely represents grazing pressure (i.e., more recently burned areas are more heavily utilized resulting in shorter structure). We selected a 2-km buffer for road and tree cover as previous research on this population found that 2-km encompassed 95% of female movements from leks to first nesting sites and therefore 2-km radius represented a realistic area to target conservation efforts [23]. For each lek we created three random points within the study area to represent available resources [23, 35]. To compare coefficients of environmental factors we calculated the z-score, which is calculated by subtracting the mean and dividing by the standard deviation [36]. We performed all analyses using program R [44].

## Results

We detected 241 lek sites during the 21-year study period ($\overset{-}{x}$ = 11.48, ±0.61 SE) and 222 leks with known fire histories were used in analyses. On average, there were 7.40 (±5.38 SE) individuals observed on each lek, but lek attendance varied from 1 to 38 birds. The number of individuals present at a lek did influence the likelihood of lek site being present the following year as there was a significant, negative relationship between lek movement and size. Larger leks were less likely to move regardless of buffer size (β_100_ = -0.04, β_250_ = -0.10, β_500_ = -0.15; all buffers p < 0.05). Despite some annual fluctuations in the number of leks and individuals detected each year, the overall trends for the study period were stable (Fig 2).

**Fig 3:**
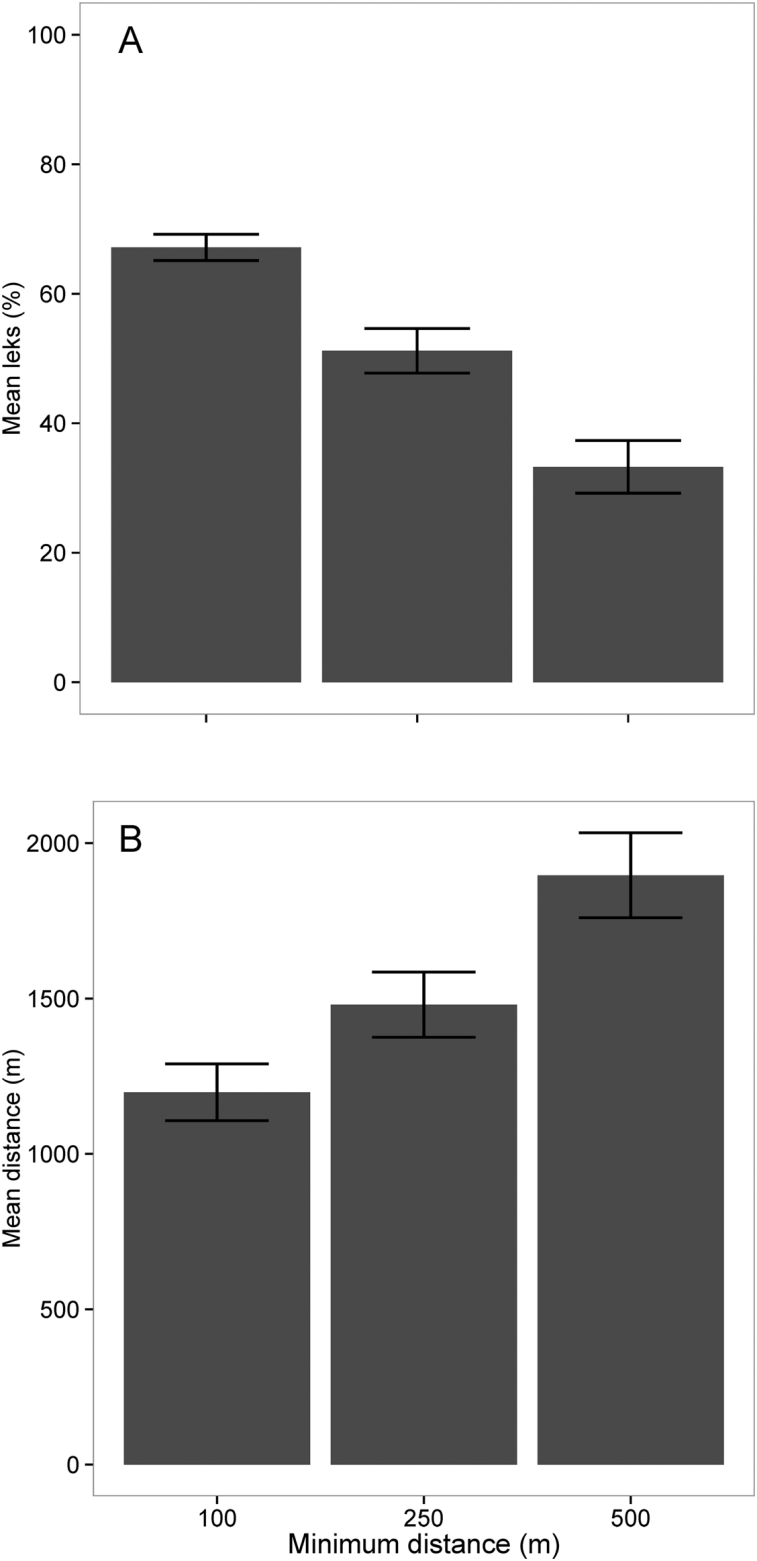
Frequency and distance of lek movement. A) Average percentage of leks that shifted and B) average distances (meters) moved each year by Greater Prairie-Chickens at the Tallgrass Prairie Preserve, OK, USA from 1991–2012. Columns represent 100, 250, and 500 meter buffers placed around lek locations to account for potential drift or observer bias. As buffer size increased the proportion of leks that shifted each year decreased, but as buffer size increased, the distance leks shifted also increased. Error bars represent standard errors.

Leks within this landscape can be characterized as dynamic, changing location from year to year. On average, each year 44.7% (±0.04 SE) of leks were in areas burned within the last year. The average distance to the closest lek of the following year was 899 m (±72.8 SE). Buffering leks resulted in a maximum annual average of nearly 65% dynamic leks, decreasing with greater buffer size (Fig 3A). Moreover, the number of leks shifting locations on an annual basis averaged 7.8 (±0.51), 6.10 (±0.52), and 4.21 (±0.55) for the 100, 250, and 500 m buffers around lek locations, respectively. Buffers also increased the average annual distance moved, with greater buffer sizes around lek locations resulting in larger distances between lek locations (Fig 3B).

**Fig 4:**
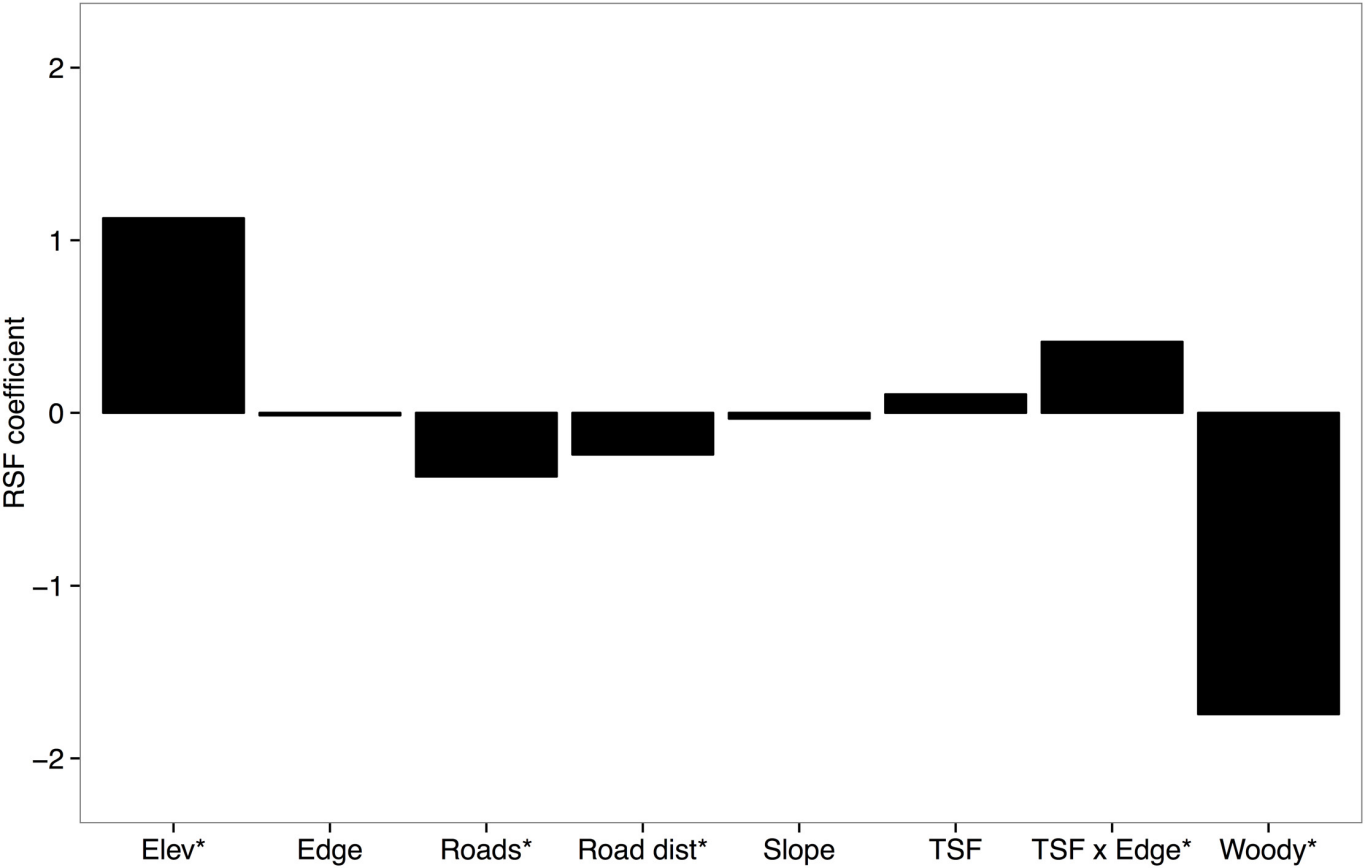
Lek site selection. Standardized coefficients from a resource selection function describing Greater Prairie-Chicken lek locations at the Tallgrass Prairie Preserve, OK, USA from 1991–2012. Bars extending upward indicate Greater Prairie-Chickens maximized use while downward bars indicate minimized use. Asterisks indicate significance at *alpha* < 0.05. Abbreviations are Elev = elevation, Road dist = distance to nearest road, TSF = time since fire, and TSF x Edge = time since fire and patch edge interaction. Refer to Table 1 for covariate definitions.

**Fig 5:**
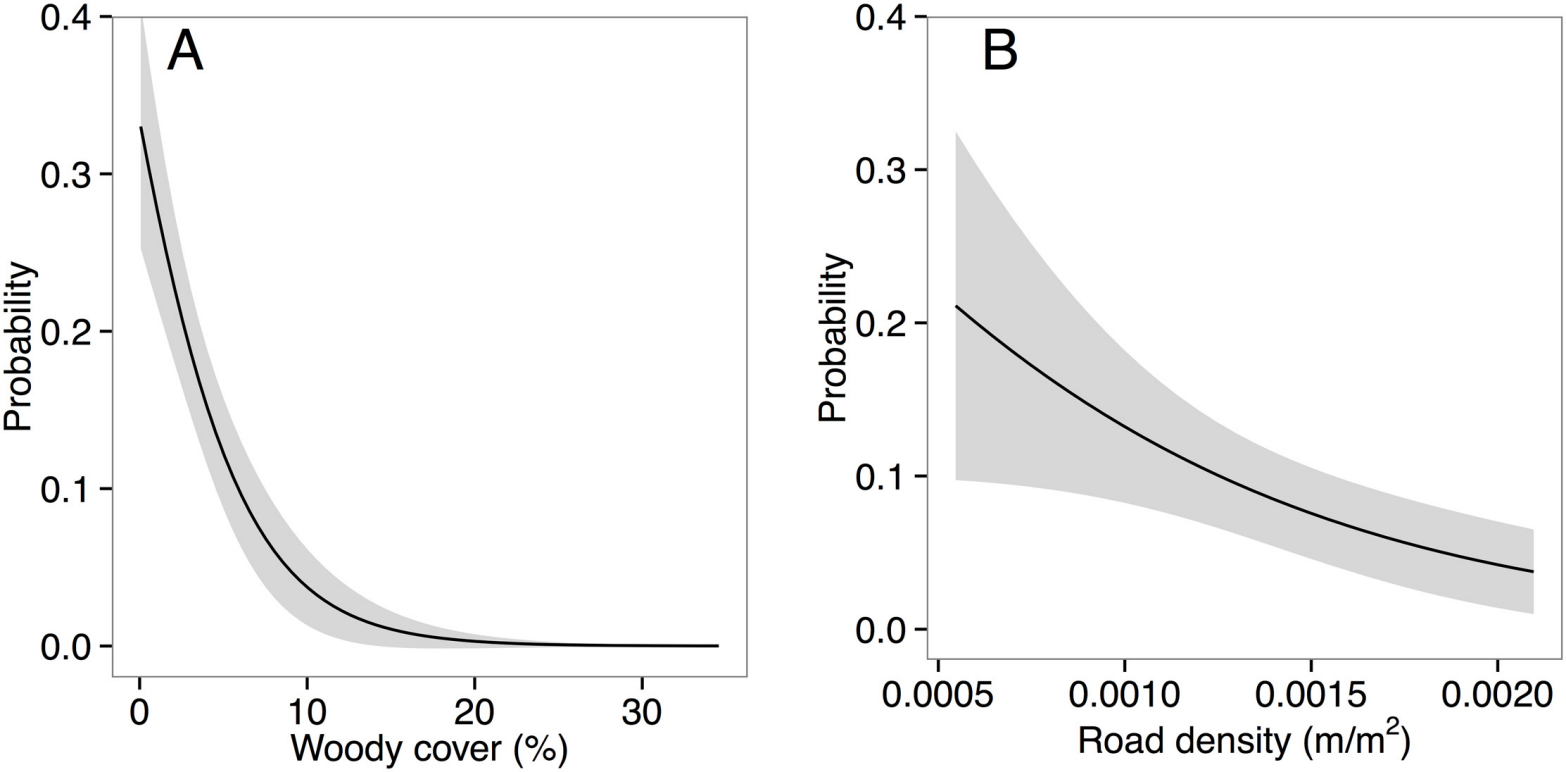
Predicted influence of tree cover and road density on lek site selection. Probability of lek site selection as a function of tree cover (A) and road density (B). Tree cover is presented as the percent cover of an area within a 2 km radius circle surrounding a lek site. Areas shaded gray represent 95% confidence intervals surrounding predicted logistic regression curves.

Lek sites were in areas that averaged 1.97 years post fire (±0.10 SE) and were 421.01 m (±0.47 SE) and 305.07 m (±18.63 SE) away from patch edges and roads, respectively (Table 1). Additionally, lek sites were in areas with low tree cover ($\overset{-}{x}$ = 1.60%, ± <1.0% SE) and road densities ($\overset{-}{x}$ = 0.001 m/m^2^, ± <0.01 SE). Lek locations were determined by a variety of environmental factors (Fig 4). In fact, as tree cover increases the probability of lek site selection declines exponentially, approaching zero at around 13% tree cover. Similarly, as road densities increase, we observed a linear decline in the probability of lek site selection which drops below 10% when roads reached densities of 0.0014 m/m^2^ (Fig 5). Lek locations were nearer to roads and at greater elevations than expected (Fig 4). The interaction of time since fire and distance to patch edge were significant in determining lek location. In recently burned areas, locations of leks were closer to patch edges; in areas with greater time since fire, locations of leks were not associated with patch edges.

**Table 1:** Covariates used in resources selection function analysis. Three random sites were selected for each lek location to determine Greater Prairie-Chicken (*Tympanuchus cupido*) lek site selection preferences.

Parameter	Mean (SE)	Range	Definition
*Random sites*			
Elevation (m)	311.89 (0.72)	257.10–354.75	Distance of lek location above sea level
Slope (%)	6.04 (0.19)	<0.01–34.81	Amount of elevation gained or lost per 100' of horizontal distance
Woody cover (%)	0.07 (<0.01)	<0.01–0.35	Area covered by trees within 2 km buffer divided by area of 2 km buffer
Road density (m/m^2^)	<0.01 (<0.01)	0.00–0.01	Length of road within 2 km buffer divided by total area of 2 km buffer
Road distance (m)	299.37 (10.34)	1.43–1307.10	Distance to nearest road (2 track or gravel, no paved roads present)
Time since fire (days)	748.48 (23.33)	7.00–3632.00	Time elapsed since a location was burned
Edge (m)	492.33 (30.07)	0.09–5807.91	Distance to nearest (fire) patch edge
*Lek sites*			
Elevation (ft)	332.29 (0.82)	300.78–358.01	
Slope (ft)	4.53 (0.23)	0.28–17.33	
Woody cover (%)	0.02 (<0.01)	<0.01–0.12	
Road cover (m/m2)	0.01 (<0.01)	<0.01–0.01	
Road distance (m)	305.07 (18.63)	2.81–1177.04	
Time since fire (days)	720.69 (37.67)	75.00–3263.00	
Edge (m)	421.01 (47.01)	1.01–4566.94	

## Discussion

Most North American grasslands lack the dynamic disturbance processes that historically created diverse vegetation structure and composition [20]. As a consequence, previous examination of prairie-chicken lek site selection has reported mostly static lek locations with temporary movements occurring in response to recruitment rates and local shifts in the abundance of males [32–34]. While these findings are accurate given their context, they may be misleading if applied broadly to all landscapes where prairie-chickens occur. Our examination of prairie-chicken lek site selection in tallgrass prairie with restored disturbance processes illustrates the dynamic nature of lek site selection patterns. We hypothesize this selection occurred as grouse tracked changes in habitat structure following disturbance from fire and grazing to maximize their ability to detect predators, attract females, and maintain nearby refugia (both for predator avoidance and female nesting cover). Although we did not measure vegetation structure directly, the effects of interacting fire and grazing on vegetation structure are well documented [18, 21, 35]. As changes in vegetation structure occur after burning and focal grazing, birds relocated lek sites to areas that are more recently disturbed and in close proximity to nearby refugia (unburned patches). Annually, approximately 45% of leks were in areas that had been burned in the previous year. Additionally, >65% of leks moved (i.e., were not at the same geographic location) on an annual basis according to our most liberal estimate (100 m buffer) while nearly 40% of leks shifted on an annual basis according to our most conservative estimate (500 m buffer) (Fig 3A). Moreover, we observed an increase in the average distance to the nearest lek from the location of a previous lek with increasing buffer size.

Previous research has offered numerous explanations for lek movement and attendance including differences in recruitment rates of males, compensatory changes in abundance of males at neighboring leks (if one lek increases an adjacent lek may decline), or changes in location of nesting habitat for females [32]. The observed changes in lek selection patterns in our study may be a combination of many of those factors, but were likely most directly affected by changes in habitat structure related to disturbance patterns across the landscape. Previous prairie-chicken research in this landscape reported leks occurring near patch edges and researchers speculated that males were selecting sites near refugia to avoid predators as leks in the center of burn patches would leave individuals vulnerable [45]. Variability in vegetation structure resulting from disturbance could allow individuals to seek locations that maximize predator detection, visibility to breeding females, and proximity to nesting cover simultaneously. As a result, our findings may be partially explained by the hot-spot hypothesis, which suggests that males establish leks in areas where they are most likely to encounter females [40], and females in this landscape most frequently select nest sites in areas that have gone >2 years without fire [23]. Therefore, evidence both for the hot-spot hypothesis and refugia from predator avoidance are supported by the significant relationship between lek site selection and an interaction of fire and patch edge—meaning that prairie-chickens are selecting sites near the edges of burned and unburned sites to lek. The selection for these locations also may alter the view-shed which could diminish long-range viewing thereby protecting prairie-chickens from aerial predators, while enhancing short range viewing which would increase visibility for females. This type of selection has been reported for Greater Sage-Grouse (*Centrocercus urophasianus*) as they attempt to minimize threats from Golden Eagles (*Aquila chrysaetos*), while maintaining close range viewing for females and the capacity to observe ground-dwelling predators [46].

In addition to the dynamic disturbance processes that influenced lek locations, many large-scale landscape components were drivers of lek selection patterns. Lek locations were characterized by areas that minimized tree cover and road densities while maximizing elevation. Similar selection patterns were reported for Greater Prairie-Chickens in Kansas [47]. The combination of these factors suggests that both predator detection and avoidance in may be influencing lek site selection patterns. For example, in grassland landscapes mesopredators are frequently associated with woody vegetation and highly fragmented areas (i.e., trees and roads), which could explain the avoidance of sites with higher road densities and trees [48]. Trees and other vertical structures are known to provide perch sites for aerial predators [49] and Lesser Prairie-Chicken (*Tympanuchus pallidicinctus*) lek persistence is negatively related to landscape-level woody vegetation [50, 51]. Additionally, many grouse species have declined in the presence of anthropogenic structures and exhibit displacement behavior and reduced survival [52, 53]. Moreover, noise from roadways can mask the sounds of approaching predators, leaving displaying individuals more susceptible to predation and reducing selection of sites in areas with high road densities [54].

Our research findings illustrate that prairie-chicken behavior is dynamic in a landscape that has spatial and temporal variability in grassland structure resulting from restored disturbance processes, and emphasizes the importance of focusing on the conservation of pattern and process to support biodiversity in rangelands [14]. Furthermore, these findings are important for future planning and targeted conservation because both counts of leks and males on leks have been used as indices of the status of prairie grouse populations [32]. Understanding the dynamic selection patterns of prairie-chickens can influence how lek count data are collected and interpreted. Previous research has suggested the permanency of leks should help to identify preferred greater prairie-chicken habitat, and this recommendation is supported in landscapes that lack dynamic disturbances or structural heterogeneity at broad scales [33]. However, our research suggests that prairie-chicken lek site selection is dynamic when disturbance regimes are restored and therefore, monitoring and interpreting data should account for this.

Adaptive monitoring programs are important as the use of restored interacting fire and grazing is becoming more common in grassland management. For example, in 2005 the Patch-burn Grazing Working Group (PBGWG) was established to discuss the use of interacting fire and grazing on private and public lands. This group is made up of members from more than 10 states that span agency employees, academics, and private landowners. While quantifying the application of patch-burn grazing throughout grasslands of the United States is extremely difficult to enumerate, it is apparent that through research and outreach, tens of thousands of acres throughout the Midwest and Great Plains have had interacting fire and grazing reinstituted. These restored landscapes are not static, and prairie-chicken management must correspondingly not be viewed as static. Recognizing the shifting mosaic of grassland structure is paramount when designing and implementing monitoring programs in these landscapes, and providing accurate inventories of sensitive species has biological, social and economic importance. Furthermore, accurate assessments of species occurrence may influence siting of future energy infrastructure in rangelands. The realization that lek locations and nesting cover move in dynamic landscapes could mean that we have greatly underestimated the amount of area used by prairie grouse in some landscapes, particularly those with interacting fire and grazing. A landscape view of dynamic habitat selection should be employed in landscapes managed at broad scales for heterogeneity when planning for conservation or development. Specifically, static buffers surrounding lek sites over a short period of time will underestimate the amount of area used by prairie-chickens in dynamic landscapes and potentially have negative population level consequences over time.
